# Dysbiosis‐Mediated Inflammation: A Pathophysiological Link Between Rheumatoid Arthritis and Periodontitis

**DOI:** 10.1111/jcpe.70063

**Published:** 2025-12-02

**Authors:** Isabel Lopez‐Oliva, Iain L. Chapple, Akshay Paropkari, Shweta Saraswat, Praveen Sharma, Stefan Serban, Paola de Pablo, Karim Raza, Andrew Filer, Thomas Dietrich, Melissa Grant, Purnima S. Kumar

**Affiliations:** ^1^ Institute of Dentistry, Barts and the London School of Medicine and Dentistry Queen Mary University of London London UK; ^2^ Periodontal Research Group, School of Dentistry, Institute of Clinical Sciences University of Birmingham Birmingham UK; ^3^ NIHR Birmingham Biomedical Research Centre in Inflammation Birmingham UK; ^4^ Birmingham Dental Hospital, Birmingham Community Healthcare NHS Foundation Trust Birmingham UK; ^5^ Clear Labs, Inc. San Carlos California USA; ^6^ James Cancer Institute and Solove Research Center The Ohio State University Columbus Ohio USA; ^7^ School of Dentistry University of Leeds Leeds UK; ^8^ Research Into Inflammatory Arthritis Centre Versus Arthritis, Institute of Inflammation and Ageing, College of Medical and Dental Sciences University of Birmingham Birmingham UK; ^9^ Department of Rheumatology Sandwell and West Birmingham NHS Trust Birmingham UK; ^10^ Department of Periodontics and Oral Medicine University of Michigan Ann Arbor Michigan USA

**Keywords:** cybernetics, dysbiosis, inflammation, pathophysiology, periodontitis, rheumatoid arthritis

## Abstract

**Aim:**

To explore mechanistic links between rheumatoid arthritis (RA) and periodontitis (PD) through the lens of subgingival microbial dysbiosis–mediated inflammation.

**Methods:**

Subgingival plaque from 100 volunteers with RA and PD (RAPD), 22 with RA (RAnoPD), 18 with PD (PDnoRA) and 19 healthy controls (noRAnoPD) was analysed using 16S‐amplicon sequencing, semi‐quantitative bead‐based flow cytometry to measure crevicular fluid cytokines and ELISA to quantify antibodies to oral pathogens and systemic inflammatory markers in serum. The RAPD group had been randomised to receive intensive non‐surgical periodontal therapy (PMPR) or oral hygiene alone and reviewed at 3 and 6 months in our previously reported study.

**Results:**

Subgingival microbial dysbiosis, as evidenced by higher species richness, alpha‐diversity and higher levels of known and putative periodontal pathobionts, was evident at baseline in RAnoPD, RAPD and PDnoRA. Higher serum antibodies to oral pathogens were recorded in RAPD. PMPR restored host–microbial homeostasis in RAPD within 3 months. Significant decreases in serum antibodies to microbial antigens and clinical measures of RA activity were seen after 3 and 6 months in the PMPR group but not controls.

**Conclusions:**

We demonstrate a mutualistic influence of RA and PD, beginning with RA‐induced dysbiosis of the periodontal microbiome, progressing to periodontal inflammation and culminating in PD‐driven exacerbation of systemic inflammation.

## 1 Introduction

Epidemiological studies strongly implicate periodontitis (PD) as an independent risk factor for rheumatoid arthritis (RA), after accounting for potential confounders such as smoking (which exacerbates both diseases), RA therapy (which can mask PD) and poor oral hygiene (due to impaired manual dexterity) (Bartold and Lopez‐Oliva 2020). The odds of RA patients exhibiting PD versus non‐RA individuals are variously reported to range between 1.82 and 20.57 (González‐Febles and Sanz 2021).

One potential mechanism underpinning the association between the two diseases is the generation of antibodies to citrullinated proteins (González‐Febles and Sanz 2021). The discovery that the oral pathobiont *Porphyromonas gingivalis* produces a peptidyl arginine deiminase (PAD) enzyme capable of citrullinating its own proteins (autocitrullination) and host proteins to generate auto‐antigens that create anti‐citrullinated protein antibodies (ACPAs) led to an avenue of research into the role of oral microbiota in the onset or exacerbation of RA (Corrêa et al. 2016). Several oral pathogens are associated with citrullination or an ACPA response (Konig et al. 2016). Gut microbiome studies have also shown that endogenous citrullination can be triggered through dysbiosis‐mediated inflammation (Möller et al. 2020). Moreover, the release of neutrophil extracellular traps (NETs) during neutrophilic inflammation in active PD forms a second major source of auto‐antigens for ACPA formation within periodontal tissues (Chapple et al. 2023).

It is now accepted that PD arises as a result of microbial dysbiosis within the periodontal crevice (Dabdoub, Ganesan, and Kumar 2016) and specific pathogens produce PADs (Curtis et al. 1999) capable of auto‐citrullination (molecular mimicry) (Wegner et al. 2010). It is also established that periodontal dysbiosis results from, and also drives, dysregulated immune‐inflammatory responses, leading to large‐scale NET release and associated hyper‐citrullinated histones (Beyer et al. 2018; Chen et al. 2022; Corrêa et al. 2019; Mikuls et al. 2018), generating autoantigens that may break immune tolerance in RA (Spengler et al. 2015). Although these lines of evidence provide a biologically plausible role for PD in RA pathophysiology, a large knowledge gap exists regarding the directionality of the PD–RA association and the role of RA in influencing the periodontal microbiome and associated oral immune‐inflammatory responses.

We hypothesised that the bi‐directional link between PD and RA is mediated through periodontal dysbiosis and resultant infection‐mediated inflammation. We tested this by combining cross‐sectional and longitudinal study data, alongside network analytical tools and machine learning algorithms trained on the microbiome dataset. Given that we have previously published the clinical outcomes of periodontal therapy in RA patients from the OPERA feasibility randomised trial (de Pablo et al. 2023), we focus here on local and systemic impacts of non‐surgical periodontal therapy at the molecular level, and not on clinical outcomes.

## 2 Materials and Methods

### 2.1 Patient Selection/Recruitment

Study volunteers were recruited as part of the OPERA (Outcomes of PEriodontal therapy in Rheumatoid Arthritis) trial, approved by South Birmingham Research Ethics Committee, UK (11/WM/0235) and registered at the ISRCTN Register (ISRCTN52833273; www.controlledtrials.com). Periodontally healthy individuals and non‐RA subjects with PD were recruited from the INSPIRED (INfluence of Successful Periodontal Intervention on REnal and Vascular Systems in patients with chronic kidney Disease) trial (15/WM/0006) (Sharma et al. 2017). Table S1 provides details of recruitment and inclusion/exclusion criteria.

### 2.2 Sample Collection

Subgingival plaque was collected in the periodontally healthy group with a curette from three representative sites (one site per sextant from the maxilla). In the PD group, samples were collected from three maxillary deep sites (PPD > 4 mm). Gingival crevicular fluid (GCF) was collected from the same sites by inserting PerioPaper strips for 30 s, and volume was measured using a Periotron 8000 and snap‐frozen in liquid nitrogen. Thirty millilitres of venous blood was collected (antecubital fossa) in serum‐separating vacutainers (E1480‐0302, Star‐Lab).

### 2.3 Microbial Sequencing

DNA was isolated as previously described (Kumar et al. 2011) and sequenced using 300‐bp paired‐end chemistry on a HiSeq‐2500 system (Illumina). Primers used for sequencing have been previously described (Kumar et al. 2011) and described in detail in Supporting Information. High‐quality sequences were assigned taxonomic identities by alignment to the HOMD database (Chen et al. 2010) using the Blastn algorithm. Analyses were conducted using QIIME (Caporaso et al. 2010) and PhyloToAST (Dabdoub, Fellows, et al. 2016).

### 2.4 Biomarkers of Periodontal Inflammation

GCF was analysed semi‐quantitatively for 27 inflammatory cytokines by multiplex bead‐based assay (Bio‐Plex Pro Human Cytokine Panel) following the manufacturer's instructions (Table S2). GCF biomarker levels were expressed as total amount per 30‐s sampling time (Lamster et al. 1985, 1986; Chapple et al. 1996, 1999).

### 2.5 Serology

Antibodies against five *P. gingivalis* antigens (peptidyl arginine deiminase [PPAD], its immunodominant citrullinated peptide [CPP3] and arginated [RPP3] epitopes, gingipain [RgpB], enolase); (Quirke et al. 2014) outer‐membrane antigens (OMA; Mikuls et al. 2012) from *P. gingivalis*, *Prevotella intermedia* and *Fusobacterium nucleatum* were measured by ELISA (Table S3). Calibration curves were derived from optical densities (ODs) to calculate the antibody concentrations, which were presented in arbitrary units per millilitre (AU/mL). When standards were unavailable, results were presented as ODs.

### 2.6 Randomisation and Treatment

Eligible patients were randomised to receive either intensive periodontal treatment or oral hygiene coaching alone. Randomisation and clinical outcomes have been previously published (de Pablo et al. 2023). Randomisation was stratified based on age, sex and ACPA status (Figure S1, CONSORT diagram). Intensive periodontal therapy consisted of full‐mouth professional mechanical plaque removal (PMPR) under local anaesthesia over 2–4 appointments by a single periodontal clinician as well as oral hygiene coaching and re‐instrumentation of all sites with probing pocket depths (PPDs) ≥ 4 mm and bleeding on probing (BOP) at 3 and 6 months' follow‐up. The control arm consisted of oral hygiene coaching (OH) alone. Patients were reviewed at 3 and 6 months post baseline, and clinical data and biological samples were acquired as described.

### 2.7 Availability of Data

All sequencing, serology and cytokine data are stored in the Ohio Supercomputing Center's Ruby Clusters and available to investigators upon request.

### 2.8 Statistical Analysis

#### 2.8.1 Microbial Data

Compositionally aware transformation (CLR) was applied to taxa‐level data. Principal coordinates analysis (PCoA) of phylogenetic (UniFrac weighted and unweighted) and non‐phylogenetic (Bray–Curtis) distance was used for dimensionality reduction. Significant differences between clusters were calculated using the Adonis and ANOSIM tests. Alpha diversity (within group) was analysed using the abundance coverage estimator (ACE) and Shannon diversity indices, and differences between group‐wise alpha diversities were measured using the Mann–Whitney *U* test. MANOVA/Wilks lambda of linear discriminant analysis (LDA) was used to test for significance. Differential abundance analysis of operational taxonomic units (OTUs) was carried out using DESeq2 and *p*‐values adjusted for multiple testing (*p* < 0.05, FDR‐adjusted Wald test). The ability of species to discriminate between groups was examined using an AI‐based machine learning algorithm (RandomForest package in R).

### 2.9 Serology

The Kolmogorov–Smirnov test was used to assess normality. Baseline antibody‐level comparisons between the four patient groups were conducted using the Kruskal–Wallis test; between‐group significance was established using Dunn's multiple comparison test (*p* < 0.05); and V1–V6 differences were assessed by the Wilcoxon test (*p* < 0.05). Correlations between levels of serum antibodies against bacterial antigens and clinical parameters were calculated using Spearman's correlation test (*p* < 0.05 and ≥ 0.75).

### 2.10 Correlation Networks

Species‐level co‐occurrence networks were created using JMP to calculate pairwise correlations. Significant co‐occurrences (defined as Spearman's rho > 0.75 and *p* < 0.05 [*t*‐test of rho]) were imported into the Python package ‘Networkx’ to create graph structures.

## 3 Results

The final accrual numbers and CONSORT workflow are shown in Figure S1.

### 3.1 Cross‐Sectional Study

Clinical data from the four patient and control groups are non‐randomised, and therefore Table 1 presents statistical significance values for between‐group differences in clinical characteristics. Participants (*n* = 159) were predominantly female, with no difference in proportions across groups. Smokers were more frequent in the RAPD group compared to the non‐RA groups (*p* < 0.05, Chi‐squared test). Ethnicity varied between study groups, while 25% of volunteers in the RAPD group lacked ethnicity information. The non‐RA groups were significantly younger than RA groups (*p* < 0.05, two‐sample *t*‐test). Within the RA population, the subgroup with periodontitis (RAPD) exhibited a higher DAS28 (disease activity score of 28 joints) score.

**TABLE 1:** Clinical characteristics of study population (*n* = 159) according to periodontal and systemic condition.

	RA with periodontitis (*n* = 100)	RA without periodontitis (*n* = 22)	Non‐RA with periodontitis (*n* = 18)	Non‐RA without periodontitis (*n* = 19)	*p*
Female proportion	72%	78%	55.6%	63%	0.2
Ethnicity					
Asian	15%	5%	11%	11%	
White	53%	90%	78%	89%	0.05
Black	6%	0%	11%	0%	
U/K	25%	5%	0%	0%	
Smoking					
Current	21%	13%	6%	5%	< 0.01
Former	30%	21%	61%	11%	
Never	49%	61%	33.%	84%	
Alcohol consumption					
Never	38%	13%	11%	15%	
1–4 times/month	37%	47%	16%	65%	0.02
1–4 times/week	19%	26%	44%	15%	
> 4 times/week	3%	13%	16.7%	5%	
Age: median (IQR)	59 (18)	60 (14)	49 (9)	36 (16)	< 0.01
BMI: median (IQR)	27 (6)	28 (3)	27 (4)	25 (5)	0.2
CumPPD: median (IQR)	86 (45)	12 (12)	111 (31)	10 (4)	< 0.01
PPD: median (IQR)	2.83 (0.86)	2.34 (0.34)	3.17 (0.96)	1.5 (0.29)	< 0.01
Median number of sites with PD > 4 mm	3.5 (5)	0	22 (28)	0	< 0.01
BOP: median (IQR)	0.19 (0.29)	0.5 (0.19)	0.38 (0.24)	0.02 (0.1)	< 0.01
CAL: median (IQR)	3.2 (1.2)	2.4 (0.64)	3.9 (1.9)	1.7 (0.24)	< 0.01
DAS28: median (IQR)	4.28 (2)	3.42 (2)	—	—	0.02
ESR: median (IQR)	14 (24)	8 (23)	—	—	0.2
VAS: median (IQR)	55 (40)	41 (24)	—	—	0.8
Tender joints: median (IQR)	7 (13)	3.5 (5)	—	—	0.2
Swollen joints: median (IQR)	1 (2)	0 (1)	—	—	0.04
ACPA status (% positive)	60.9%	54%	—	—	0.6

*Note*: RA data not available in systemically healthy controls (—).

Abbreviations: ACPA, anti‐citrullinated protein antibodies measured with anti‐CCP (cyclic citrullinated peptide) tests; BOP, bleeding on probing; CAL, clinical attachment level; CumPPD, cumulative probing depth; DAS, disease activity score; ESR, erythrocyte sedimentation rate; IQR, inter‐quartile range; *n*, number of patients per group; PPD, probing pocket depth; RA, rheumatoid arthritis; U/K, unknown; VAS, visual analogue score.

^a^ Chi‐square test (or Fisher's exact test where appropriate). Between‐group comparisons were made using *t*‐tests, Mann–Whitney *U* or Chi‐squared tests, as appropriate.

### 3.2 Intervention Study

Detailed comparison of clinical characteristics between RAPD groups with and without intervention has been reported previously (OPERA; de Pablo et al. 2023). Briefly and for context, PMPR was associated with reduced cumulative probing depths, periodontal inflamed surface area, DAS28 and joint ultrasound scores versus OHI (oral hygiene index) alone (controls).

#### 3.2.1 Distinct Subgingival Microbial Assemblages Are Evident in RA

**FIGURE 1:**
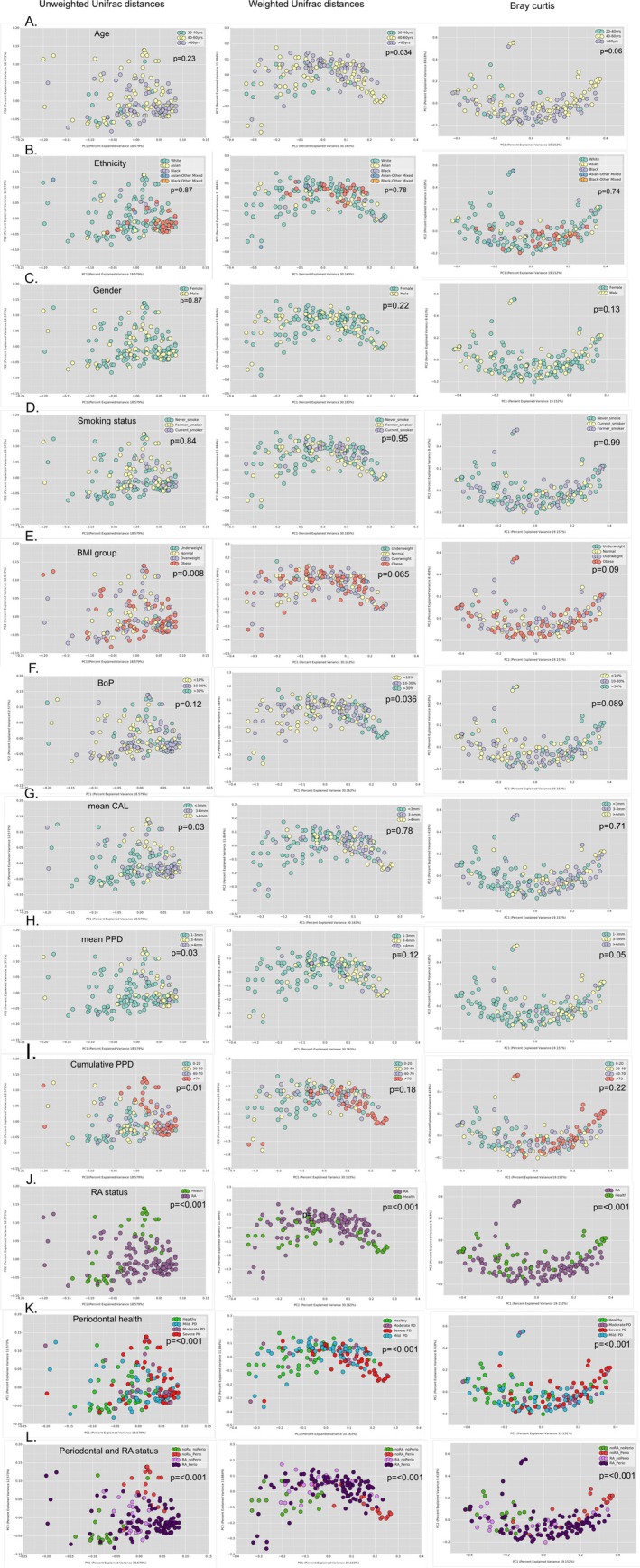
Patient‐ and subject‐level determinants of subgingival microbial assemblages. Principal coordinate analysis of branch‐length (weighted and unweighted UniFrac distances) and phylotypic (Bray–Curtis dissimilarity matrix) metrics of CLR‐transformed taxa are shown based on age decades, ethnicity, gender, smoking habits, BMI, bleeding on probing (BOP), mean clinical attachment level (CAL), mean periodontal probing depth (PPD) and cumulative PPD (*n* = 160). Significance of clustering was tested with the analysis of similarity (ANOSIM, *p* < 0.05).

**FIGURE 2:**
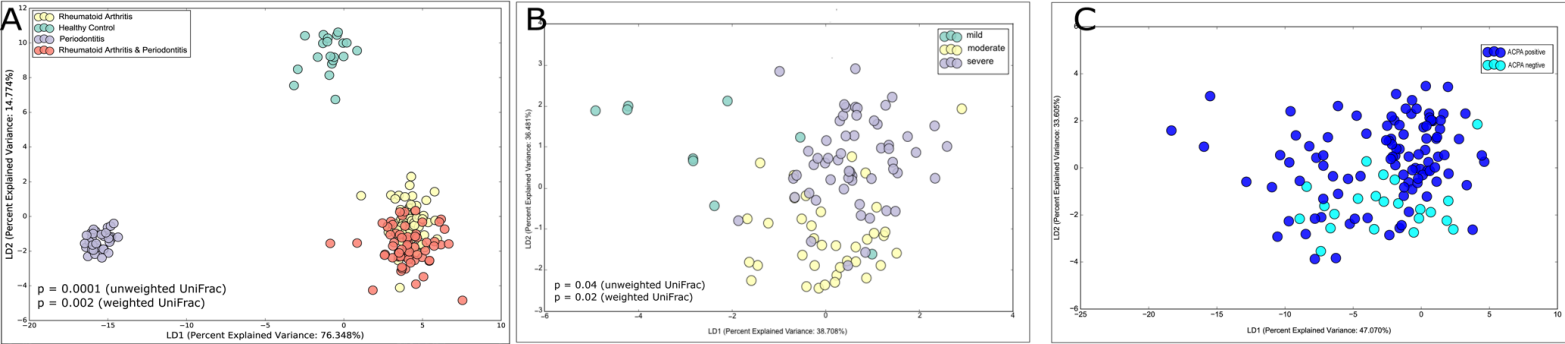
Relative impact of rheumatoid arthritis and periodontitis on subgingival microbial assemblages. Linear discriminant analysis of CLR‐transformed taxa relative abundances of species‐level operational taxonomic units (s‐OTUs) are mapped on periodontal disease and rheumatoid arthritis in panel A and on DAS‐28 score in panel B. Significance of clustering was tested using Dunn's test with joint ranks.

RA status and severity of PD emerged as the most consistent drivers of microbial similarity using branch‐length (weighted and unweighted UniFrac distances) and phylotype (Bray–Curtis dissimilarity matrix) (Figure 1, *p* < 0.001, PERMANOVA). Unlike systemically healthy patients, patients with RA could not be separated based on their periodontal health status (Figure 2A, *p* > 0.05, Dunn's test with joint ranks). Furthermore, the microbial signatures of individuals with RA clustered significantly based on disease activity score (DAS28) and ACPA positivity (Figure 2B,C, *p* < 0.001, Dunn's test with joint ranks). Finally, a machine learning algorithm trained on this dataset was able to predict RA with an out‐of‐box error rate of 4.05%, while the error rate for PD was 38.6% (Figure S2). Interestingly, none of the microbial community parameters (alpha diversity) or selected taxa ( *P. gingivalis* , *F. nucleatum* and *Tannerella forsythia* ) showed robust and/or significant correlations with periodontal clinical parameters such as BOP, PPD and CAL.

#### 3.2.2 The RA‐Influenced Subgingival Microbiome Is Highly Networked

**FIGURE 3:**
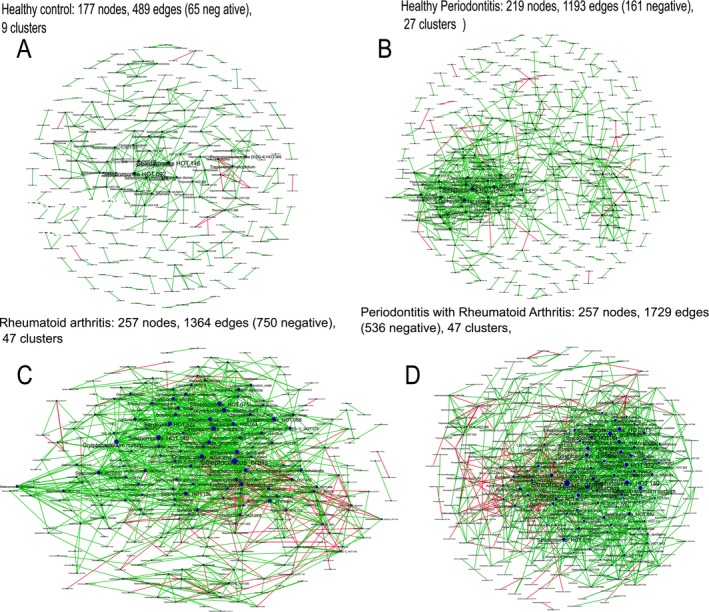
Co‐occurrence network of bacteria. Systemically and periodontally healthy controls are shown in (A), periodontitis in (B), rheumatoid arthritis in (C) and rheumatoid arthritis with periodontitis in (D). Each network graph contains nodes (circles sized by relative abundance per group) and edges (lines). Nodes represent species‐level OTUs and edges represent Spearman's *ρ*. Edges are coloured green for positive correlation and red for negative correlation. Only significant correlations (*p* < 0.05, *t*‐test) with a coefficient of at least 0.75 are shown.

**FIGURE 4:**
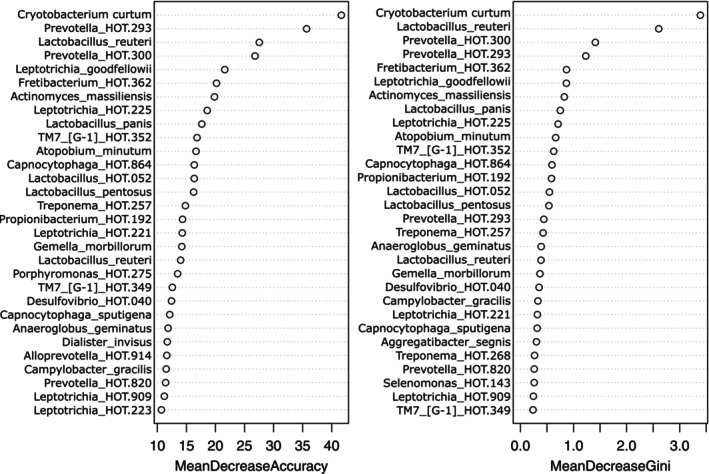
Prediction capabilities of microbial taxa. Random forest classifier was used to validate the findings of the discriminant analysis. The mean decrease in accuracy indicates the reduction in prediction accuracy when each variable is removed (A), while the mean decrease in Gini coefficient defines the role of the variable in creating pure splits in the decision tree (B). Thus, variables with higher accuracy as well as higher Gini coefficients are the most important.

**FIGURE 5:**
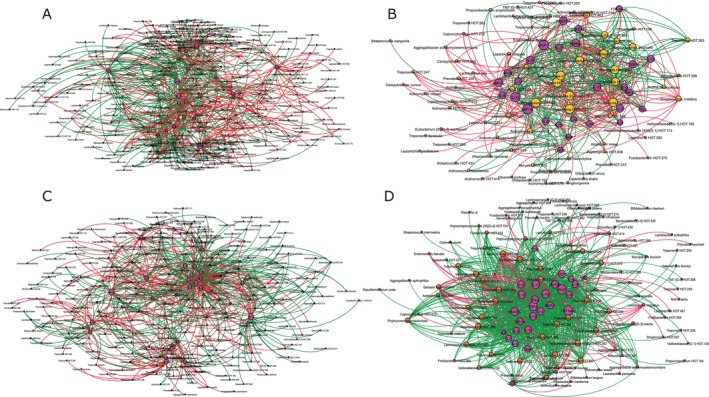
Effect of scaling and root debridement in resetting immune‐microbial networks. Co‐occurrence networks of cytokines, chemokines and bacteria in the periodontal pocket are shown. Panel A and C are baseline networks, while panel B shows re‐establishment of networks following oral hygiene instructions and panel D shows re‐establishment of networks following scaling and root debridement. Each network graph contains nodes (circles sized by relative abundance per group) and edges (lines). Nodes represent species‐level OTUs and edges represent Spearman's *ρ*. Edges are coloured green for positive correlation and red for negative correlation. Only significant correlations (*p* < 0.05, *t*‐test) with a coefficient of at least 0.75 are shown.

The microbiome in RA showed greater species richness and alpha diversity and higher levels of gram‐positive facultative species compared to non‐RA (Figure S3, *p* < 0.001, Dunn's test with joint ranks); however, no differences could be detected between RA patients with or without PD. Species richness (Chao and ACE indices) and alpha diversity (Shannon index) also showed low correlations with mean plaque scores (Spearman's *ρ* = 0.23, 0.19 and 0.26 respectively, *p* > 0.05), further supporting the hypothesis that factors other than poor oral hygiene contribute to the observed dysbiosis. Network analysis revealed highly connected dense inter‐bacterial networks in RA patients with PD, with progressively lower connectivity in RA without PD, PD without RA and periodontal and systemically healthy individuals (Figure 3). *Cryptobacterium curtum* , as well as species belonging to the genera *Leptotrichia*, *Prevotella*, *Lactobacillus*, *Actinomyces* and *TM7‐G1*, emerged as important discriminants in the RA group microbiome (Figure 4). We found strong (≥ |0.8|) and statistically significant (*p* < 0.05, Spearman's rho) correlations between these discriminant species and IL‐1β, IL‐2, IL‐12, IL‐13, IL‐17A, MIP‐1β, RANTES, GM‐CSF and PDGF‐β (Figure 5A,C). Notably, *C. curtum* was correlated to a high number of pro‐inflammatory cytokines including TNF‐α, IL‐8, IP‐10 and MIP‐1α, and *P. gingivalis* correlated with IL‐6, TNF‐α and IL‐1β, while *T. forsythia* negatively correlated with IL‐9, MIP‐1α, MIP‐1β and TNF‐α.

#### 3.2.3 Periodontal Dysbiosis Associates With Higher Serum Antibody Levels to Oral Pathogens

RA patients with and without PD showed significantly higher serum antibody levels to PPAD (*p* = 0.0013), *Pg*‐enolase (*p* = 0.0031) and RPP3 (*p* = 0.001) compared to non‐RA controls (with and without PD). Serum antibodies to outer membrane proteins (OMPs) of *P. gingivalis* (*Pg*‐OMP) and *P. intermedia* (*Pi*‐OMP) were similarly elevated in RA patients with and without PD. Although a trend towards a greater antibody response to *Pg*‐antigens was observed with increasing PD severity, only anti‐*Pg*‐enolase and PPAD antibodies were significantly higher in PD patients versus controls (PG‐Eno *p* = 0.002; PPAD *p* = 0.001). Serum anti‐*Pg* antibodies also showed a weak but statistically significant correlation with the relative abundance of subgingival *P. gingivalis* (*R* = 0.33, *p* < 0.0001). By contrast, antibodies to *F. nucleatum* OMPs were elevated in patients with PD (with and without RA) compared to periodontally healthy volunteers with and without RA. Weak, but statistically significant positive correlations were observed between anti‐*Pg* OMA antibodies and citrullinated proteins (C‐TNC5 [*R* = 0.287, *p* = 0.002], CCP [*R* = 0.215, *p* = 0.023]).

#### 3.2.4 Periodontal Disease Severity Strongly Correlates With RA Activity

**FIGURE 6:**
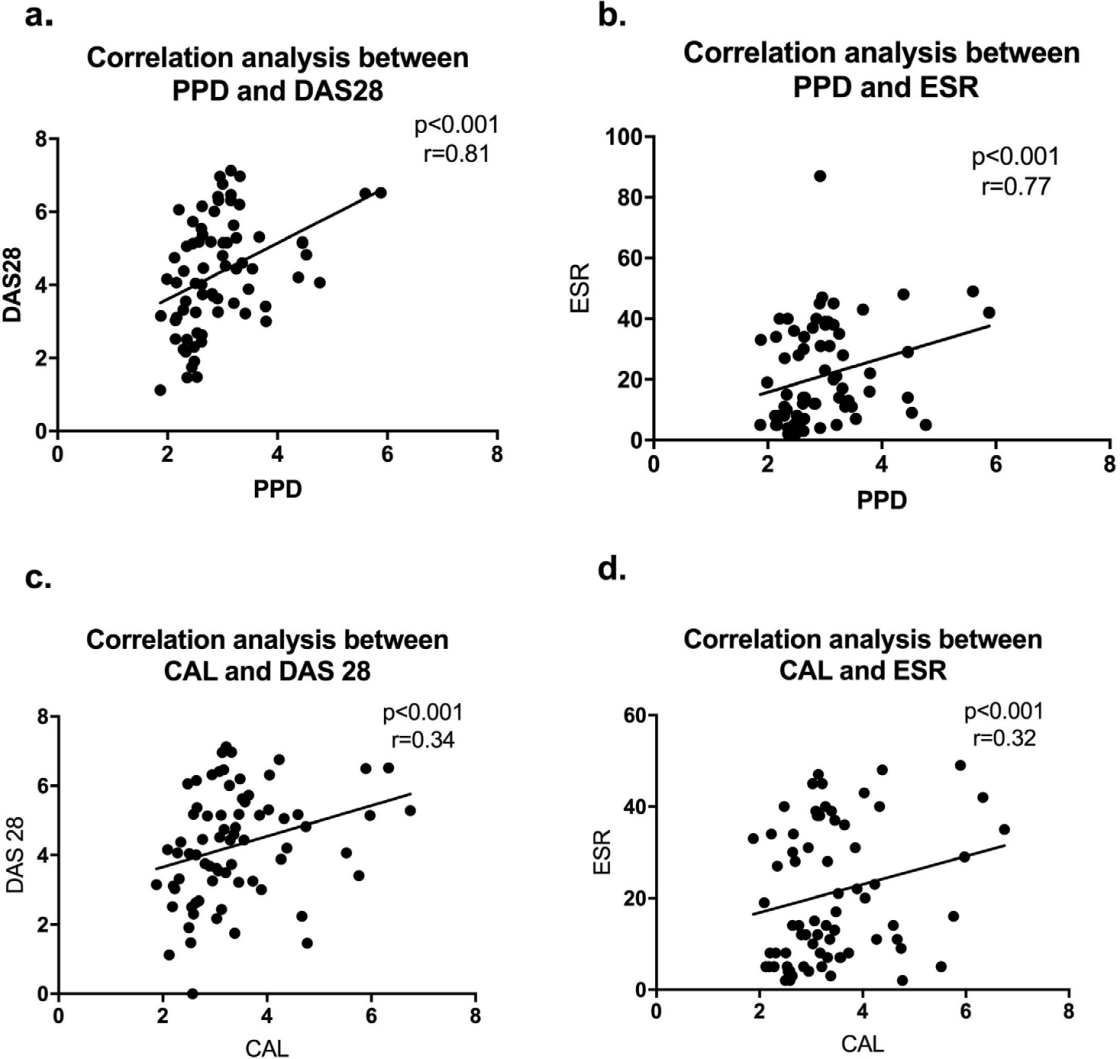
Correlation analysis between periodontal and RA clinical parameters. Spearman's correlation (significance *p* < 0.05). CAL, clinical attachment loss; DAS28, disease activity score of 28 joints; ESR, erythrocyte sedimentation rate.

PPD and CAL were significantly correlated with DAS28, ESR, number of swollen joints, number of tender joints and visual analogue pain scale (VAS) related to RA disease activity (*p* < 0.05, Spearman rank correlation test, Figure 6).

#### 3.2.5 Intensive Non‐Surgical Periodontal Therapy Re‐Instates Subgingival Host–Microbial Networks

We have previously reported that PMPR resulted in significant improvement of periodontal clinical parameters in all individuals when compared to controls. There was a significant decrease of IL‐1β, IL‐8, IL‐9, IL‐10, IL‐12, VEGF, TNF‐α and IL‐15 (*p* < 0.05, repeated‐measures ANOVA) in GCF in treated ACPA‐positive RA compared to the control group.

Forty‐two OTUs changed significantly post PMPR. Species belonging to the genera *Fretibacterium*, *Treponema*, *Tannerella* and *Veillonellaceae* decreased significantly following PMPR, while species of the genera *Streptococcus*, *Actinomyces* and *Neisseria* increased at 3 months post intervention. Ten OTUs were identified only at baseline and not at 3 months. Changes in the levels of these 42 OTUs did not show correlations with change in PPD, CAL or BOP (*r* ^2^ = 0.18–0.23, *p* > 0.05).

However, when pre‐ and post‐treatment bacterial–cytokine interaction networks were compared, PMPR resulted in increased numbers of host–microbial interactions (measured by the number of network connections or ‘edges’ between cytokines and bacteria at baseline and 6 months post intervention) as well as ‘re‐wiring’ of several interactions (evidenced by larger numbers of bacterial species anchored by each cytokine hub, as well as the weight of these edges [or strength of correlation]). At baseline, more inter‐bacterial than cytokine–microbial connections were evident in both groups (892 inter‐bacterial edges vs. 501 cytokine–bacterial edges in the intervention group and 1105 vs. 670 in the control group, Figures 3 and 5). Following periodontal therapy, cytokine–bacterial edges demonstrated a five‐fold increase, with no change in the control group (Figure 5A–D). Nodes anchored by IL‐1β, IL‐4, IL‐6, IL‐10, IL‐13, MIP‐1β and PDGF‐β showed the greatest re‐wiring (measured by the D‐n score) with the addition of new species to the hubs.

#### 3.2.6 Intensive Non‐Surgical Periodontal Therapy Decreases Circulating Antibodies to Oral Bacteria

PMPR led to reductions in antibodies to OMAs that were significant for anti‐FN levels (mean −14.92, *p* = 0.026) and anti‐PI (mean −4.52, *p* = 0.009) but not significant for anti‐*Pg* (mean −85, *p* = 0.25). During the course of the study, 17% and 20% of the intervention and control groups, respectively, reported changes in RA medications (*p* = NS).

#### 3.2.7 Appreciable Reductions in Clinical RA Scores Are Evident Following Intensive Non‐Surgical Periodontal Therapy

Clinical indicators of RA activity improved in both cohorts, with more pronounced improvement in the intervention group (*p* < 0.05), particularly 3 months post operation. The control group showed a higher tender joint count and patient global VAS score, whereas all components of disease activity improved in the intervention group (DAS28, CRP, ESR, tender joints, swollen joints). This difference in improvement between groups could not be attributed to changes in RA medication.

## 4 Discussion

The link between RA and PD is reported as bi‐directional (de Pablo et al. 2009), each disease impacting the other (Hussain et al. 2020). However, cybernetic modelling supports circular causality. The traditional concept of linear relationships is based on reciprocal theory, that is, exposure A leads to outcome B and B leads to A. However, cybernetics envisions systems where relationships are both recursive and reciprocal, that is, two events have mutual influence upon each other, rather than a simple unidimensional cause‐and‐effect relationship (Bateson and Donaldson 1991). Here, we found a mutual influence of RA and PD, beginning with a RA‐influenced dysbiosis of the periodontal microbiome, leading to periodontal inflammation, and PD‐influenced exacerbation of systemic inflammation, which is reduced following periodontal therapy to lower dysbiosis‐induced inflammation. While linear theory implies that intervention can be effective only at the beginning of each causal factor (i.e., early RA or PD), circular theory suggests that intervention at any time in the process can break the cycle of cyclical amplification. Our data support this by demonstrating improvements in molecular and clinical measures post periodontal intervention in patients already under treatment for RA.

The reported data corroborate and build upon our previous discovery that RA has a significant impact on the periodontal microbiome (Lopez‐Oliva et al. 2018). The first line of evidence is that RA emerged as the most important determinant of periodontal microbial composition, superseding local factors such as BOP, deep PPDs and CAL. Consistent with this, microbial profiles within the RA group clustered by DAS28 score and ACPA positivity, irrespective of periodontal status, while species richness and diversity did not correlate with dental plaque biofilm levels. Furthermore, large intergeneric networks were observed only in the two RA groups. In contrast to congeneric hubs (comprised of evolutionarily similar species), intergeneric hubs are created either by ecosystem characteristics that support organisms with similar growth and nutritional requirements or by shared metabolic or structural interactions between bacteria (Sfenthourakis et al. 2006). Finally, a machine learning classifier trained on the subgingival microbiome predicted RA cases more accurately than PD. Taken together with our previous study, these data suggest that systemic inflammation due to RA is an early driver of subgingival dysbiosis and that microbial assemblages created during a state of periodontal health persist in established disease. This observation is in contrast to the impact of hyperglycaemia (Ganesan et al. 2017) or smoking (Mason et al. 2015) on this ecosystem, where the systemic/environmental factor causes an initial dysbiosis in the health‐compatible microbiome, followed by further compositional shifts upon PD onset. It is possible that the systemic inflammatory burden imposed by RA overwhelms the PD‐associated inflammation in terms of habitat filtering, and hence PD may not further destabilise the subgingival microbiome.

As previously reported, *C. curtum* , a gram‐positive, assacharolytic, anaerobic rod that degrades arginine to citrulline, ornithine and ammonia, continues to emerge as a key community member of the RA‐subgingival microbiome. In the present study, not only was this species identified at high levels in over 80% of RA patients with PD, but it also emerged as a network anchor of a large intergeneric hub. Moreover, levels of these species correlated positively with several pro‐inflammatory cytokines in the GCF. This species is poorly studied, having undergone several taxonomic and evolutionary re‐classifications, and is a candidate for further investigation as an oral biomarker for RA severity.

Interestingly, several other species, notably those belonging to the genera *Leptotrichia*, *Prevotella* and *Actinomyces*, also emerged as key members of the RA‐microbial community. Recent studies have reported enrichment of *Prevotellae* in subgingival, salivary and faecal samples of individuals at high risk for RA as well as those with new‐onset RA (Wells et al. 2020). *Prevotellae* bind to terminal galactose residues on the Fc‐region of IgG, leading to increased levels of circulatory and tissue agalactosylated or hypogalactosylated IgG (Ogrendik et al. 2005). Higher levels of agalactosylated and hypogalactosylated IgG strongly correlate with RA frequency and severity (Firestein et al. 2012). *Leptotrichia* and *Actinomyces* have previously been identified as taxa that characterise new‐onset RA irrespective of periodontal status (Scher et al. 2016). Our data also corroborate the association between *P. gingivalis* and RA, as evidenced by appreciably higher levels of circulating antibodies to *P. gingivalis* antigens in RA patients with and without PD; however, the organism was identified in only 40% of individuals with both PD and RA and 3% of periodontally healthy individuals with RA.

Taken together, the data point to multiple mechanisms by which dysbiosis‐mediated inflammation may contribute to RA initiation and exacerbation, for example, protein citrullination, generation of other neo‐autoantigens such as neutrophil extracellular traps (NETs), loss of immune tolerance through molecular mimicry and bystander activation of the immune system.

We also found evidence to support our initial hypothesis that the systemic impact of PD is attributable to community‐mediated inflammation rather than specific species within the periodontal microbiome. For instance, serum levels of anti‐OMA antibodies from various species were elevated in RA with and without PD and correlated significantly with RA‐specific clinical and protein markers. There is a need for further studies using global approaches to characterise the proteomic profile of the serum microbiota in RA patients.

Network graph theory forms the basis of social network platforms where the number of connections (edges) between individuals (nodes) and the type (friendly, neutral or antagonistic) and strength of exchanges in the different edges are used to measure the popularity of individuals. Nodes with abundant edges and frequent interactions become ‘community influencers’ and coalesce to form ‘hubs’. In biological terms, influential nodes and hubs are likely anchors of the community and control the flow of resources to the system. RA patients with and without PD showed tightly woven hubs of certain anaerobic bacteria, indicating an important role in controlling the flow of resources in the disease microbiome. However, few and sparsely connected hubs were observed between these bacteria and pro‐ or anti‐inflammatory cytokines/chemokines. This is a significant finding, since the acquisition of microbiota into an ecosystem is normally driven by their interactions with the local immune system; and in patients with RA, this local interaction appears to be overwhelmed, possibly by systemic inflammation. We have previously observed similar network topography in hyperglycaemic individuals (Kumar et al. 2020). Taken together, it suggests that the sustained selection pressure exerted by systemic inflammation creates an enriched microbiome lacking the diversity necessary to maintain balanced immune–microbial alliances. Such a breakdown in mutualism has been proposed to account for the dramatic increase in autoimmune and inflammation‐based disorders, especially in developed nations (Zheng et al. 2020), suggesting that the link between RA and PD is not merely bi‐directional, but circular.

In order to discern whether sub‐optimal immune–microbial interactions can be optimised in the continued presence of the systemic pressor, we employed differential network analysis to extract interactions that are unique to each time, a process called network re‐wiring. The host–bacterial network showed an exponential increase in connections and hubs anchored by both pro‐ and anti‐inflammatory cytokines, providing evidence that non‐surgical periodontal therapy resets mucosal immune–microbial interactions within 3 months, enabling the immune system to regain control of a dysregulated partnership. However, the system continues to evolve in its metastable state over 6 months, indicating the critical need for continued 3‐monthly supportive care to sustain the fragile immune–microbial alliance. Non‐surgical therapy also appears to offer systemic benefits, such as the reduction in circulating antibodies to microbial antigens.

One limitation of our study is that patients were sourced and funded from two different studies, which contributed to differences in group sizes and age distributions. However, as is seen in Table 1, patients included in the study showed generalised periodontal destruction. Importantly, although there were notable differences between groups in terms of smoking status, age and ethnicity, these factors do not appear to account for the observed differences in the microbiome. Additionally, smoking prevalence was unexpectedly low in our RA cohorts, and did not align with the pathogenic microbiome profiles identified. A further limitation is the absence of a formal sample size calculation, which reflects the pilot and feasibility design of the OPERA clinical trial.

The observed differences in the RA‐associated oral microbiome may be influenced by clinical characteristics such as age or RA medication use. However, within our study, none of these parameters appeared to account for the microbial shifts beyond periodontal and rheumatological status (Figure S3). It is possible that non‐RA medications or unrecorded comorbidities may have influenced the oral microbiota. Given the lack of comprehensive data on these factors, we cannot exclude their potential impact, and future studies are warranted to explore their role more thoroughly.

In summary, we report evidence of a circular relationship between RA and PD, beginning with an RA‐influenced dysbiosis in the subgingival microbiome during a state of periodontal health, that remains in established PD. Systemic inflammation in RA is accompanied by higher periodontal inflammation, circulating antibodies to periodontal pathogens and a correlation between PD severity and RA disease activity. Periodontal treatment reinstates immune–microbial interactions, reduces antibody levels to circulating microbial antigens and improves systemic markers of RA. This not only points to the importance of integrating periodontal care in the management of RA but also serves to increase awareness of the impact of the inflammatory burden imposed by RA on periodontal management in RA. However, long‐term studies employing larger sample sizes and comprehensive omics approaches are needed to expand upon these observations.

## Author Contributions

Study design: Isabel Lopez‐Oliva, Iain L. Chapple, Thomas Dietrich, Melissa Grant and Purnima S. Kumar. Clinical study: Isabel Lopez‐Oliva, Iain Chapple, Praveen Sharma, Stefan Serban, Paola de Pablo, Karim Raza, Andrew Filer, Thomas Dietrich and Melissa Grant. Sample and data analysis: Isabel Lopez‐Oliva, Akshay Paropkari, Shweta Saraswat and Purnima S. Kumar. Manuscript writing: Isabel Lopez‐Oliva, Iain Chapple, Akshay Paropkari, Shweta Saraswat, Praveen Sharma, Stefan Serban, Paola de Pablo, Karim Raza, Andrew Filer, Thomas Dietrich, Melissa Grant and Purnima S. Kumar.

## Funding

This work was supported by the National Institute of Dental and Craniofacial Research (R01 DE022579), the Department of Health, Social Services and Public Safety, UK Government, the National Cancer Institute (U01 CA188250) and the Department of Health, Social Services and Public Safety, UK Government (PB‐PG‐0609‐19100, DRF‐2014‐07‐109, PDF‐2014‐07‐055).

## Conflicts of Interest

The authors declare no conflicts of interest.

## Supporting information


**Data S1:** jcpe70063‐sup‐0001‐Supinfo.pdf.

## Data Availability

The data that support the findings of this study are available from the corresponding author upon reasonable request.
