# Supplementary material for: Dysbiosis‐Mediated Inflammation: A Pathophysiological Link Between Rheumatoid Arthritis and Periodontitis
Source: J Clin Periodontol. 2025 Dec 2;53(3):466–77. doi: 10.1111/jcpe.70063 (PMC12890453; doi:10.1111/jcpe.70063)
Supplement: Supplementary file 1 — Data S1: jcpe70063‐sup‐0001‐Supinfo.pdf. [file JCPE-53-466-s001.pdf]

METHODS

1. PATIENTS WITH RHEUMATOID ARTHRITIS: THE OPERA STUDY

The OPERA study was funded by the National Institute of Health Research (NIHR) (Grant Reference Number PB-PG-0609-19100). The trial was registered at the ISRCTN Register (registration number ISRCTN52833273; www.controlled-trials.com).

Recruitment: Patients diagnosed with Rheumatoid Arthritis (according to the revised 1987 ACR criteria) from three different hospitals in Birmingham (Queen Elisabeth Hospital, Heartlands Hospital and City and Sandwell Hospital) were contacted and informed about the study and if interested in taking part, were invited to the screening appointment.

Screening appointment: The screening appointment started with an assessment of the general health using a detailed medical questionnaire and taking the blood pressure, weight and height of the patient.

The assessment was followed by an examination of the swelling and pain (tenderness) of 28 joints according to the disease activity score (DAS) 28. For the assessment of periodontal status, a Periodontal Pocket Chart was performed recording the clinical attachment level, bleeding on probing, pocket depth and gingival recession. To guarantee consistency, the same operator (I.L.O.) performed the periodontal examination at baseline and in the review visits of the longitudinal part of the trial. Covariate data was collected at baseline such as age, gender, ethnicity, socio-economic status, education, marital status, smoking habits, alcohol consumption, comorbidities and medication.

Inclusion and exclusion criteria

The main purpose of these criteria was to assure a sufficient disease activity for both RA and periodontitis (Table 1). To that end, a compilation of validated questionnaires were given to patients, disease activity score (DAS28) was calculated, and full dental and periodontal assessment was performed during the screening visit. The main inclusion criteria had the aim to assure enough RA and periodontitis activity, which standardized through a DAS 28 higher than 3.2, and moderate to severe periodontal disease. To include patients with a sufficient level of inflammation, cumulative probing depth higher than 40 mm was defined as inclusion criteria, this being the sum of the highest probing depth per tooth when probing depths were higher or equal to 4 mm. The purpose of the exclusion criteria was to assure all patients started the study with stable medication (treatment with DMARD for >3 months and stable dose for >2 months). The exclusion criteria aimed to reject patients with other types of periodontal disease and rheumatic or systemic autoimmune disease other than RA. Also patients that had underwent periodontal treatment (within 12 prior to baseline), surgical procedures (within 12 weeks prior to baseline) or glucocorticoids injections (within 4 weeks prior to baseline) were excluded from the study.

Supplemental Table 1: Inclusion and exclusion criteria for the OPERA (Outcomes of Periodontal Therapy in Rheumatoid Arthritis) study. Abbreviations: RA, Rheumatoid Arthritis; ACR, American College of Rheumatology; DAS, Disease Activity Score; DMARD, Disease Modifying Anti-Rheumatic Drug; CAL, Clinical Attachment Loss.

Inclusion	Exclusion
Age 18+, accepting to participate in the study and willing to sign a consent form	Other rheumatic autoimmune diseases different from RA, or significant systemic involvement secondary to RA
Diagnosed according to the revised 1987 ACR criteria for the classification of rheumatoid arthritis (RA)	Other inflammatory joint disease (past or current) different from RA
DAS28 ≥3.2	Juvenile idiopathic arthritis (JIA) or juvenile rheumatoid arthritis (JRA) and/or diagnosis of RA before the age of 16

DAS28 >5.1 if biologics drugs being administrated	Any surgical procedure, including bone/joint surgery/synovectomy (including joint fusion or replacement) within 12 weeks before included in the study or scheduled to be administrated during study or Intra-articular or parenteral glucocorticoids within 4 weeks before commencement of the study
Treatment with DMARD for ≥ 3 months and stable dose for ≥ 2 months	Significant concomitant disease, which would impede patient participation in the investigators opinion
Generalized moderate to severe chronic periodontitis as evidenced by pocketing with clinical attachment loss (CAL>=4 mm on at least 2 non-adjacent teeth AND cumulative probing depth>=40mm). Cumulative pocket depth is the sum of the deepest probing depths of at least 4mm on each tooth	Periodontal treatment received within 12 months of the inclusion in the study or other dental problems contraindicate the participation in the study

Randomisation: Patients that qualified for the intervention study according to the inclusion/exclusion criteria were offered a verbal and written explanation of the study and a consent form (Patient Information Sheet -Treatment, and Informed Consent Form - Treatment). If willing, patients were randomized into one of the two treatment arms, stratified by their ACPA status. Randomization was performed through the Birmingham Clinical Trials Unit, which used a web-based system developed by the unit. Investigators were blinded from the group allocation for the duration of trial.

2. SYSTEMICALLY HEALTHY VOLUNTEERS: THE INSPIRED STUDY Approval for this study was obtained from the South Birmingham Research Ethics Committee, UK (Approval codes: Inspired - 15/WM/0006) and the study was funded by the NIHR (Grant Reference Number: DRF-2014-07-109). Biological samples from systemically healthy volunteers were obtained as part of the INSPIRED feasibility study (Influence of Successful Periodontal Intervention on Renal and Vascular Systems in patients with Chronic Kidney Disease)

Recruitment: A total of 40 systemically healthy patients were recruited as part of the INSPIRED feasibility study from the periodontal and oral surgery department of Birmingham Dental Hospital as well as non-dentist/hygienist staff from within the hospital.

Screening appointment: The screening appointment started with a first assessment of the general health in a detailed medical questionnaire and by taking the blood pressure, weight and height. Covariate data was collected at baseline such as age, gender, ethnicity, socio-economic status, education, marital status, smoking habits, alcohol consumption, comorbidities and medication. For the assessment of periodontal status, a Periodontal Pocket Chart was performed recording the clinical attachment level, bleeding on probing, pocket depth and gingival recession.

Inclusion/exclusion criteria: To participate in the systemically healthy group (for both periodontitis and periodontally healthy groups), patients were excluded if they suffered from any self-reported systemic illness including hypertension or diabetes or if they had received periodontal treatment within the last year.

-Periodontitis group: the inclusion criteria was to be diagnosed with moderate to severe periodontitis, defined as Clinical attachment level (CAL) >4mm in at least 2 non-adjacent teeth and

cumulative probing depth>40 mm. Cumulative pocket depth is the sum of the deepest probing depths of at least 4mm on each tooth.

-Periodontally healthy group: the specific exclusion criteria for periodontal health was probing depth of >4mm at any site.

3. MULTIPLEX ASSAY FOR CYTOKINE QUANTIFICATION Gingival crevicular fluid (GCF) was analyzed for the semi-quantification of inflammatory cytokines. Once PerioPaper strips were thawed, all 4 strips from each patient were pooled into a single cryotube and eluted by adding 200 µl of elution buffer containing BSA 0.5% and 1x PBS and incubated for 1 hour at room temperature. 200 µl of the eluted samples were then aliquoted into 96 well masterplates. Cytokine analysis was performed using a commercially available multiplex bead-based assay designed to quantitate multiple cytokines (Bio-Plex Pro™ Human Cytokine Panel, 27-Plex from Bio-Rad™ Platform) following manufacturer's instructions. A panel containing 27 cytokines was selected (Table 3). Magnetic beads were diluted to a 1x concentration in Bio-Plex assay buffer and were vortexed for 30 seconds. After pre-wetting the plate with Bio-Plex buffer (100µl), beads (50µl) were added into each well and incubated for 30 minutes at RT. To wash the plates, wash buffer (150 µl) was added to each well and plates were attached in the magnetic holder for 30 seconds. After flicking out the liquid, plates were detached from the magnetic holder and wash buffer was again added. This process was repeated 3 times in every wash step. Standards were prepared as 1 in 8 dilutions. Samples and standards (50 µl) were added in the plates and incubated for 30 minutes, covering the plate with sealing aluminum foil at RT on a shaker (Luckham R11/TW, speed 2). After repeating the washing step, detection antibody (25 µl) was added in each well in a 1x concentration (diluted in detection antibody diluent) and incubated for 30 minutes, covering the plate with sealing aluminum foil at RT on a shaker. After repeating the wash step, streptavidin-phycoerythrin reporter (50 µl) was added and incubated for 10 minutes. After a final wash step, beads were suspended in assay buffer (125 µl) and shaken for 30 seconds. Fluorescence was detected on a Luminex 100/200 (USA).

Supplemental Table 2. Cytokines analyzed with the Bio-Plex Pro™ Human Cytokine Panel assay. Abbreviations: IL, interleukin; FGF, Fibroblast growth factor; GM-CSF, Granulocyte-macrophage colony-stimulating factor; G-CSF, Granulocyte colony-stimulating factor; MIP, Macrophage inflammatory protein; RANTES, regulated upon activation, normal T cell expressed and secreted; TNF, tumour necrosis factor; PDGF-BB, Platelet-Derived Growth Factor-BB ; MCP, Monocyte chemoattractant protein.

**Analyte**	**Bead location**	**Analyte**	**Bead location**	**Analyte**	**Bead location**
IL-1β	39	IL-10	56	IFN-γ	21
IL-1ra	25	IL-12(p70)	75	IP-10	48
IL-2	38	IL-13	51	MCP-1 (MCAF)	53
IL-4	52	IL-15	73	MIP-1α	55
IL-5	33	IL-17A	76	MIP-1β	18
IL-6	19	Eotaxin	43	PDGF-BB	47
IL-7	74	FGF basic	44	RANTES	37

IL-8	54	G-CSF	57	TNF-α	36
IL-9	77	GM-CSF	34	VEGFs	45

4. DETECTION OF ANTIBODIES AGAINST *P. GINGIVALIS* PROTEINS

Antibodies against five *P. gingivalis* –antigens were measured by ELISA (Suplemmentary Table 3): whole PPAD and immunodominant epitope 3 (citrullinated and arginated), gingipain (RgpB) and P. gingivalis –enolase. The sequences of the reactive epitopes have been described previously (Quirke et al., 2013) (Montgomery, Venables, & Fisher, 2013). Patient sera with high concetration of antibodies were selected as positive controls to create calibration curves for each plate following 7 2-fold dilutions. However, no appropriate controls were found for arginated enolase, fibrinogen, arginated tenascin, anti-RPP3 and PPAD.

Supplemental Table 3

Peptide	Sequence	Stock concentration	Coating concentration	Supplier
PPAD	CATATC-GGTACC-TGAAAAAGCTTTTACAGGCTAAA GCCTTGATTC	7mg/ml	10µg/ml	Oxford University
CPP3	CAKTDSYWT-Cit DYTGWFAMYDC	5mg/ml	10µg/ml	Oxford University
RgpB	RgpB-6xHis	0.81mg/ml	5 µg/ml	Jagiellonian University
PG-enolase	ckiig-X-eilds-X-gnptvec	1 mg/ml	10µg/ml	Oxford University

5. DETECTION OF ANTIBODIES AGAINST MEMBRANE PROTEINS FROM P. GINGIVALIS, FUSOBACTERIUM NUCLEATUM AND TANERELLA FORSYTHIA

To investigate whether the immune response is specifically targeted against *P.gingivalis* antigens and not targeted against any of the other periodontal pathogens, outer membrane antigens (OMAs) antibodies against other key periodontal pathogens *(P. gingivalis, Fusobacterium Nucleatum and Tanerella Forsythia)* were also investigated as previously described. Calibration curves were derived from optical densities to calculate antibody concentration, which were presented in arbitrary units per mL (AU/mL). When standards were not available, results were presented as optical density units (ODs). Raw data were processed using Graphpad Prism 7.0 (GraphPad Software Inc, California, USA).

Statistical tests were computed using SPSS 23 (IBM Statistics). Kolmogorov-Smirnov test was used to assess normality. Baseline antibody level comparisons between the four groups of patients were conducted using the Kruskal-Wallis test and significance between each group comparison was calculated using Dunn's multiple comparison test (significance $p<0.05$). Differences between proportions of patients were tested using Pearson's Chi-squared test. Differences between antibody levels at baseline (V1) and at 6 months post-intervention (V2) in each group were assessed using Wilcoxon test (significance at $p<0.05$). Correlations between levels of serum antibodies against bacterial antigens

and clinical parameters, were calculated performing the Spearman's correlation test ($p<0.05$ and ≥ 0.75) in JMP (SAS Institute Inc.).

6. MICROBIOME ANALYSIS

Subgingival plaque samples (n=281) were sequenced from the OPERA and INSPIRED participants according to availability of the subgingival plaque sample. 17 patients were discarded due to drop-outs or not enough clinical data available, resulting in a total number of patients included of 264. Subgingival samples from two types of sites (deep pockets and shallow pockets) were selected from each patient in the systemically healthy with periodontitis group (n=17) to compare the two locations. To investigate the effect of periodontal therapy in RA, plaque samples were collected from the two randomized groups (intervention and control) after 3 and 6 months after randomization (n=240).

DNA isolation: Bacterial DNA was extracted from the plaque samples in the research laboratories of the Ohio State University School of Dentistry. Plaque samples collected with currettes were diluted in PBS (200µl) and centrifuged (8000 rpm for 1 minute) to remove the Tris-buffered saline and other salts. Plaque samples were collected with paper-points, 200 µl of the solution was added to the vial containing 0.25 g glass bead and beat for 60 seconds at 500 rpm twice followed by centrifuging (1400 rpm) for 2 minutes. Supernatant was carefully aspirated and placed in a labelled sterile 1.5 ml tube. Bacterial DNA was isolated using Qiagen DNA MiniAmp® kit (Qiagen, Valencia, CA, USA) and protocol manufacturer instructions were followed.

Sequencing: Library preparation and sequencing (the reading of the nucleotides present in DNA) and library preparation was conducted in the Mr DNA laboratories was performed on a MiSeq platform (Mrdnalab, Shallowater, TX, USA) following the manufacturer's instructions (**Error! Reference source not found.**). PCR primers of the V1-V3 (spanning E.coli 16S gene regions 8-27 and 519-536) and V7-V9 (spanning E.coli 16S gene regions 1099-1114 and 1528-1541) of the 16S rRNA gene were used.

Sequence data pre-processing: Once the sequences of DNA have been generated, Phylogenetic Tools for Analysis of Species-level Taxa (PhyloToAST) version 1.4.0rc1 and Quantitative Insights Into Microbial Ecology (Qiime) version 1.9.1 pipelines were used to pre-process the sequences to reduce errors and bias produced in the sequencing. Quality control of 98% was required for adaptor sequences and sequences of less than 300bp were discarded. Chimeric sequences were depleted using ChimeraSlayer (v. 1.9.0, identify_chimeric_seqs.py). All quality filtered sequences were aggregated (97% similarity) and de novo operational taxonomic units (OTUs) were identified using HOMD database using the UCLUST65 method.After merging the two primers, the number of species assigned to an OTU by both primers was reduced by half to prevent doubling sequences picked by both primers. Primer averaging was carried out as previously described. To parse the OTU-sequence data, first any OTUs that occurred in less than 5% percent of samples and/or OTUs that make up less than 0.01% of the overall sequences were removed. The removed OTUs are separated in a file for future examination. For the following steps, a representative sequence was picked from the database for each OTU (instead of operating with the entire dataset). Finally, taxonomic identity was assigned to each OTU by alignment of the representative sequence to the HOMD database using the Blastn algorithm.

Microbiome data analysis: Rarefaction analysis was performed to confirm if saturation was reached for all the samples, using the alpha_rarefaction.py script (Qiime). Compositionally aware transformation (CLR) was applied to taxa-level data Beta diversity (between-group) was investigated performing Principal coordinates analysis (PCoA) using both distances matrices: phylogenetic (UniFrac weighted and unweighted) and non-phylogenetic (Bray–Curtis). Significant differences between clusters was calculated using the Adonis and ANOSIM tests. PCoA plots were generated by the python package PhyloToAST using PCoA.py script. Alpha diversity (within group) was analyzed using Abundance Coverage Estimator (ACE) and Shannon diversity using alpha_diversity.py script, and differences between group-wise alpha diversities was measured using Mann-Whitney U test. Phylogenetic trees were created with iTOL (http://itol.embl.de/, version 3.4.1). The core species in each group were identified using Qiime's script (core_microbiome.py) when species were present in at least 80% of the patients in each group. Differences

between species relative abundance in each group were calculated using Deseq (differential_abundance.py) and significance was obtained from adjusted p values for multiple comparisons (FDR-adjusted Wald Test). This function uses a negative binomial distribution of raw counts to estimate between-group differences, while accounting for sampling effort (library size) and dispersion of each category (taxon or functional gene). Network correlations were investigated to test for differences in co-occurrence patterns between microbial communities from different ecosystems. Significant correlations pairwise were calculated using Spearman's correlation ($p<0.05$ and $\rho \geq 0.75$) in JMP (SAS Institute Inc.) and inserted in Python (Networkx package to create the graph structures and visualized in Gephi (https://gephi.org/). Network anchor OTUs were defined as significant different abundance between the 2 groups, high betweenness centrality (top 20%) and belonging to the core microbiome of each group.

RESULTS

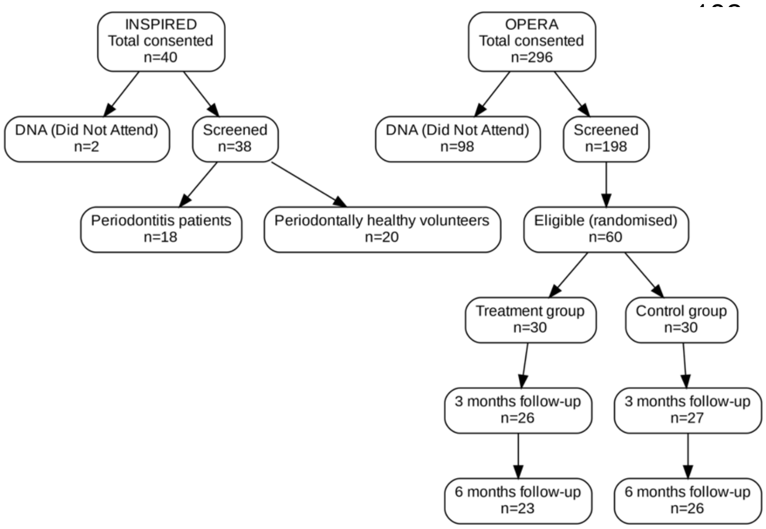

Supplemental Figure 1, CONSORT Flow Diagram of enrolment, intervention allocation and follow-up in the OPERA and INSPIRED (systemically healthy arm) studies. -Periodontitis group: defined as Clinical attachment level (CAL) >4mm in at least 2 non-adjacent teeth and cumulative probing depth>40 mm (Cumulative pocket depth is the sum of the deepest probing depths of at least 4mm on each tooth).-Periodontally healthy group: probing depth of <4mm at any site.

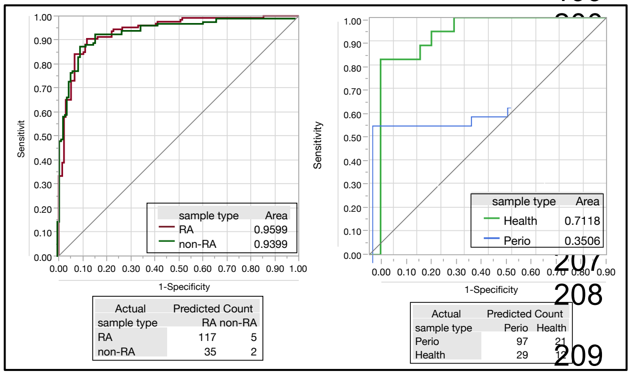

Supplemental Figure 2. Receiver operator curves of RandomForest classifier curves of a RA and Periodontal Disease obtained when using a Random Forest classifier to predict the subgingival microbial signatures of individuals. The machine learning algorithm was able to predict RA with an out-of-box error rate of 4.05%, while the error rate for periodontitis was 38.6%

Supplemental Figure 3. Characteristics of RA-influenced microbiome. Kernel plot of density curves for alpha diversity between the 4 groups; subjects with and without Rheumatoid Arthritis (RA), with and without periodontitis using matrices Chao 1 (A.) and Shannon index (B.). The peaks of each kernel plot indicate the median values for each group, and the x-axis shows the data range. Significant pairwise differences are denoted by same alphabets on top of bars ($p < 0.05$, Wilcoxon signed rank test). Figure C

represents the differences in abundance of species by gram staining and oxygen requirement characteristics.

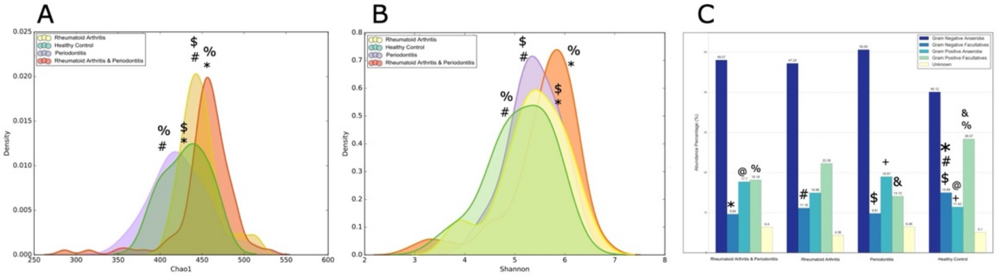

Supplemental Table 4. Grouping parameters tested for significance in clustering the oral microbiome. Test=ANOSIM, significance if p<0.05. Abbreviations: BoP, bleeding on probing; CAL, clinical attachment level; RA, rheumatoid arthritis; PPD, pocket probing depth.

Grouping factor	Groups	ANOSIM significance
**Periodontal classification**	Eke&page 2012	Significant
**BoP**	Mean <10% vs 10-40% vs >40%	Significant
**CAL**	Mean <3mm, 3-4mm, >4mm	Significant
**RA**	RA vs systemically healthy	Significant
**PPD**	Mean 1-3 mm vs 3-4 mm vs >4 mm	Not significant
**Age**	20-40years old vs 40-60 vs >60 years old	Not significant
**Gender**	Male vs female	Not significant
**Smoking status**	Never vs current vs former	Not significant

Figure 3. PCoA of unweighted (a) and weighted (b) Unifrac distances in RAPD patients grouped by type of medication. n =31. Test: ANOSIM, significance if p<0.05. Abbreviations: RA, rheumatoid arthritis; PD, chronic periodontitis, DMARDs, Disease-modifying anti-rheumatic drugs.

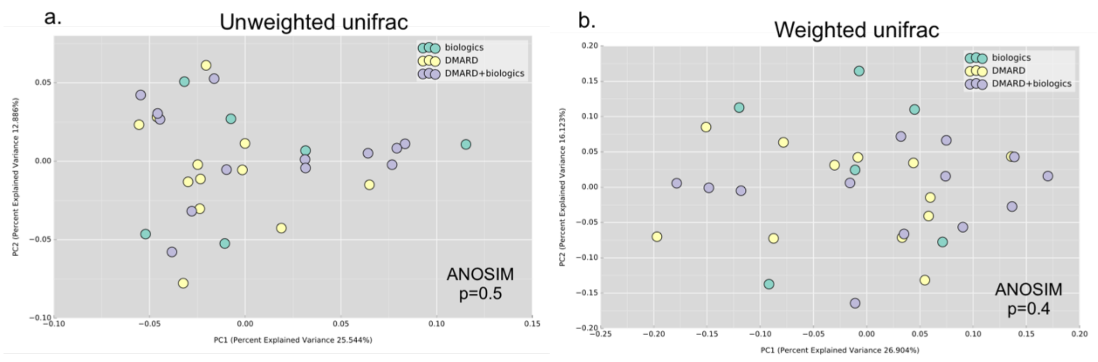

Montgomery, A. B., Venables, P. J., & Fisher, B. A. (2013). The case for measuring antibodies to specific citrullinated antigens. *Expert review of clinical immunology, 9*(12), 1185-1192.
Quirke, A.-M., Lugli, E. B., Wegner, N., Hamilton, B. C., Charles, P., Chowdhury, M., . . . Culshaw, S. (2013). Heightened immune response to autocitrullinated Porphyromonas gingivalis peptidylarginine deiminase: a potential mechanism for breaching immunologic tolerance in rheumatoid arthritis. *Ann Rheum Dis*, annrheumdis-2012-202726.
